# Off‐Label Initiation of Gabapentin and Valproic Acid Among Long‐Stay Nursing Home Residents With and Without Dementia

**DOI:** 10.1111/jgs.70574

**Published:** 2026-07-03

**Authors:** Lauren B. Gerlach, Lan Zhang, Ana Montoya, Sarah L. Krein, Andrew R. Zullo, Julie P. W. Bynum, Donovan Maust

**Affiliations:** ^1^ Department of Psychiatry University of Michigan Medical School Ann Arbor Michigan USA; ^2^ Institute for Healthcare Policy and Innovation, University of Michigan Ann Arbor Michigan USA; ^3^ Department of Internal Medicine University of Michigan Medical School Ann Arbor Michigan USA; ^4^ Center for Clinical Management Research, VA Ann Arbor Health Care System Ann Arbor Michigan USA; ^5^ Department of Epidemiology Brown University School of Public Health Providence Rhode Island USA; ^6^ Transformative Health Systems Research to Improve Veteran Equity and Independence Center of Innovation, VA Providence Health Care System Providence Rhode Island USA

## 1 Introduction

Antiseizure medication prescribing in nursing homes (NHs) has increased substantially over the past decade, particularly among residents with Alzheimer's disease and related dementias (ADRD), largely driven by gabapentin and valproic acid [1]. Although appropriate for seizure disorders and other Food & Drug Administration (FDA)‐approved indications, these medications are frequently used off‐label for symptoms common in NHs, including non‐neuropathic pain and behavioral symptoms of dementia [1]. Off‐label use is not inherently inappropriate; however, evidence supporting gabapentin and valproic acid for off‐label indications is limited, while harms—including sedation, falls, cognitive impairment—are well documented [2, 3]. Quantifying potentially off‐label initiation can help clinicians and policymakers distinguish evidence‐based treatment from potentially low‐value prescribing.

## 2 Methods

We conducted a cross‐sectional study using 2021 national Medicare fee‐for‐service and Medicare Advantage claims linked with Minimum Data Set (MDS) assessments. The cohort included long‐stay NH residents aged 66 years or older with at least 100 days in a NH and no more than 30 days outside the facility. Medication initiation was defined as a Part D prescription fill for gabapentin or valproic acid with no fill for the same medication in the prior 6 months; residents could have prior use of other antiseizure medications.

Potential indications were identified from ICD‐10 diagnosis codes in the 6 months before and after the index fill, a window selected to capture diagnoses documented shortly before or after prescribing. FDA‐approved indications included seizure disorders for both medications, postherpetic neuralgia for gabapentin, and bipolar disorder or migraine for valproic acid. Initiations without these diagnoses were classified as potentially off‐label.

Among off‐label gabapentin users, we characterized neuropathic and non‐neuropathic pain diagnoses. Among off‐label valproic acid initiators with ADRD, behavioral symptoms were identified using the MDS Distressed Behavior in Dementia Indicator (DBDI > 0; range 0–18, higher scores indicate more frequent or severe behaviors), assessed over a 7‐day look‐back period [4]. ADRD was identified using a validated claims algorithm supplemented with MDS indicators [5]. The University of Michigan Institutional Review Board approved this study with waiver of informed consent.

## 3 Results

Among 40,808 gabapentin initiators, 34,688 (85.0%) had no FDA‐approved indication (Table 1). Off‐label gabapentin initiation was common among residents with and without ADRD (Figure 1; 83.4% vs. 86.7%). Among off‐label gabapentin initiators, 60.2% had both neuropathic and non‐neuropathic pain diagnoses, 36.9% had non‐neuropathic pain only, 1.1% had neuropathic pain only, and 1.9% had neither.

**TABLE 1:** Characteristics of long‐stay nursing home residents initiating gabapentin or valproic acid, by presence of an FDA‐approved indication.

Characteristics	Gabapentin			Valproic acid		
	FDA‐approved (*N* = 6120)	Off‐label (*N* = 34,668)	Std diff	FDA‐approved (*N* = 10,412)	Off‐label (*N* = 19,173)	Std diff
Overall, %	15.0	85.0		35.2	64.8	
Resident‐level						
Age at index date, years						
65–74	2584 (42.2)	10,388 (30.0)	0.32	4062 (39.0)	3633 (19.0)	0.55
75–84	2273 (37.1)	12,646 (36.5)		4133 (39.7)	7569 (39.5)	
85+	1263 (20.6)	11,654 (33.6)		2217 (21.3)	7971 (41.6)	
Sex						
Female	3676 (60.1)	22,183 (64.0)	0.15	6105 (58.6)	12,126 (63.3)	0.10
Race/ethnicity						
Non‐Hispanic White	4579 (74.8)	28,102 (81.0)	0.17	8101 (77.8)	16,210 (84.6)	0.20
Non‐Hispanic Black	1197 (19.6)	4742 (13.7)		1689 (16.2)	2042 (10.7)	
Hispanic	143 (2.3)	686 (2.0)		259 (2.5)	318 (1.7)	
Other	201 (3.3)	1158 (3.3)		363 (3.5)	603 (3.2)	
Rurality						
Urban	5389 (88.1)	29,638 (85.4)	0.11	9198 (88.3)	16,327 (85.2)	0.14
Clinical diagnoses						
Dementia	3592 (58.7)	18,098 (52.2)	0.13	8595 (82.6)	17,447 (91.0)	0.25
Depression	4154 (67.9)	21,415 (61.7)	0.12	7434 (71.4)	12,941 (67.5)	0.08
Anxiety	3358 (54.9)	17,271 (49.8)	0.10	6982 (67.1)	12,096 (63.1)	0.09
Bipolar	756 (12.4)	2315 (6.7)	0.20	3891 (37.4)	312 (1.6)	1.01
Schizophrenia/psychosis	1402 (22.9)	5570 (16.1)	0.17	4677 (44.9)	6207 (32.4)	0.26
Pain	5986 (97.8)	33,890 (97.7)	0.003	9988 (95.9)	18,049 (94.1)	0.08
Neuropathic pain	3530 (57.7)	18,435 (53.2)	0.09	3476 (33.4)	5087 (26.5)	0.15
Non‐neuropathic pain	5864 (95.8)	33,158 (95.6)	0.002	9921 (95.3)	17,895 (93.3)	0.09
Baseline medication use						
Benzodiazepine	1415 (23.1)	6724 (19.4)	0.09	3553 (34.1)	6324 (33.0)	0.03
Antipsychotic	1500 (24.5)	6406 (18.5)	0.15	4955 (47.6)	7565 (39.5)	0.18
Opioid	2668 (43.6)	15,296 (44.1)	0.01	3163 (30.4)	5407 (28.2)	0.05
Antiepileptic	3929 (64.2)	8532 (24.6)	0.88	5205 (50.0)	4514 (23.5)	0.59
Antidepressant	3764 (61.5)	20,016 (57.7)	0.07	7133 (68.5)	13,671 (71.3)	0.06
Memory drug	988 (16.1)	5327 (15.4)	0.02	3263 (31.3)	7689 (40.1)	0.18
Cognitive function scale						
1	198 (3.2)	1340 (3.9)	0.20	2195 (21.1)	2298 (12.0)	0.30
2	2533 (41.4)	16,402 (47.3)		2605 (25.0)	4168 (21.7)	
3	1652 (27.0)	9192 (26.5)		4189 (40.2)	10,135 (52.9)	
4	1428 (23.3)	6860 (19.8)		980 (9.4)	1716 (9.0)	
Disruptive behavior	442 (7.2)	2408 (6.9)	0.02	2064 (19.8)	5475 (28.6)	0.21
Facility‐level						
Staffing level						
> 4.1 h (recommended)	2021 (33.0)	11,135 (32.1)	0.09	3823 (36.7)	6686 (34.9)	0.03
3–4.1 h (marginal)	3268 (53.4)	17,959 (51.8)		5309 (51.0)	9755 (50.9)	
< 3 h (substandard)	831 (13.6)	5594 (16.1)		1280 (12.3)	2723 (14.3)	
Profit status						
Non‐profit	1333 (21.8)	9586 (27.6)	0.18	1944 (18.7)	5183 (27.0)	0.20
For‐profit	4769 (77.9)	24,971 (72.0)		8433 (81.0)	13,954 (72.8)	
Bed size						
Small (< 100 beds)	1760 (28.8)	11,726 (33.8)	0.09	3380 (32.5)	7147 (37.3)	0.11
Medium (100–140)	2122 (34.7)	11,552 (33.3)		3482 (33.4)	6260 (32.7)	
Large (140+)	2238 (36.6)	11,410 (32.9)		3550 (34.1)	5766 (30.1)	
Dementia care unit	1019 (16.7)	6072 (17.5)	0.13	1919 (18.7)	4374 (22.8)	0.12

*Note:* Resident characteristics were measured during the 12‐month baseline period before medication initiation. Pain was identified using claims‐based diagnostic codes for chronic or acute pain conditions. Non‐neuropathic pain excluded neuropathic diagnoses including neuralgia/neuritis, radiculopathy, diabetic neuropathy, polyneuropathy, and herpes zoster. The Cognitive Function Scale is derived from MDS assessments and categorizes cognitive impairment from 1 (intact cognition) to 4 (severe impairment). Distressed behavior was defined using the MDS Disruptive Behavior in Dementia Indication (DBDI > 0), reflecting behavioral symptoms such as aggression, agitation, or other disruptive behaviors. Facility staffing level reflects total nursing hours per resident day. Standardized differences (Std Diff) were calculated to compare characteristics between residents initiating medication with and without FDA‐approved indications; values > 0.10 indicate meaningful differences.

**FIGURE 1:**
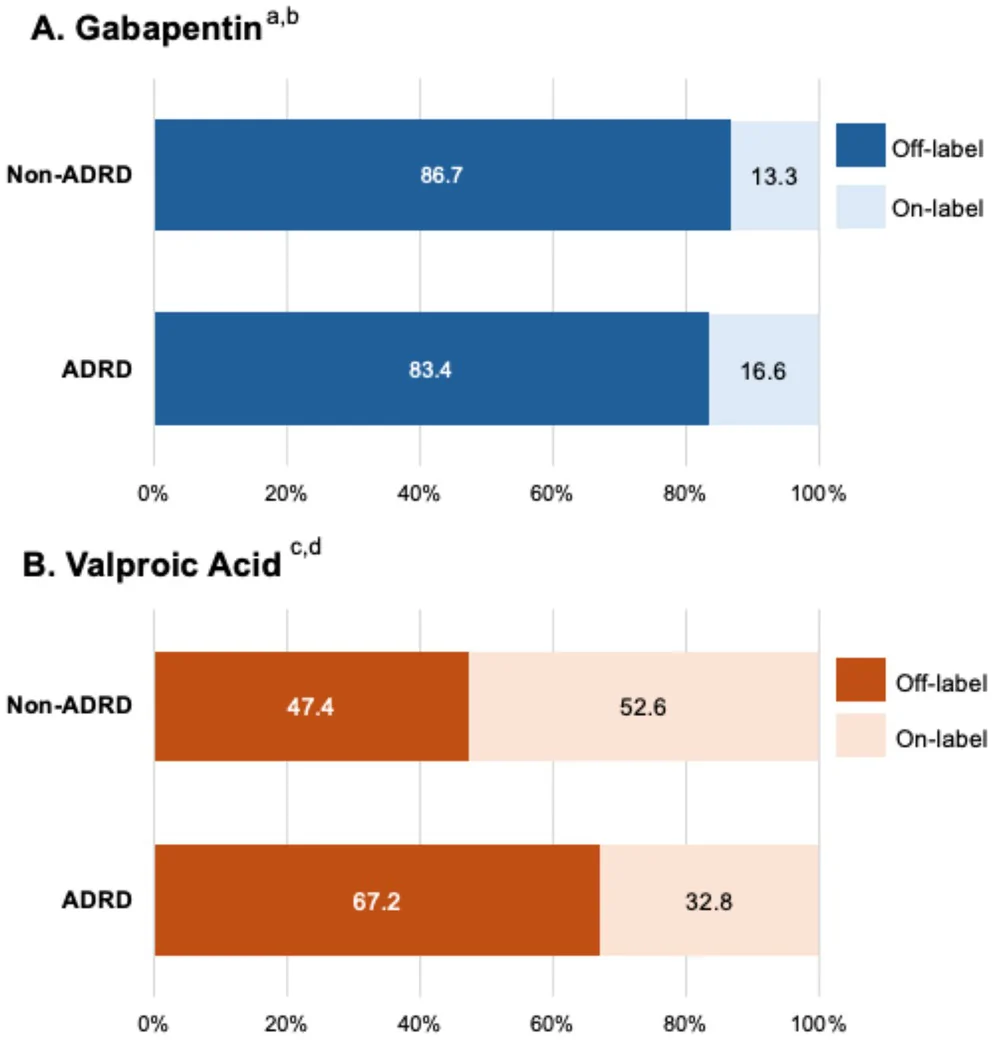
Potentially off‐label versus FDA‐approved initiation of gabapentin and valproic acid among long‐stay nursing home residents, by dementia status. Bars show the proportion of medication initiators with an FDA‐approved indication versus no documented FDA‐approved indication, stratified by Alzheimer's disease and related dementias (ADRD) status. ^a^Gabapentin, residents without ADRD. FDA‐approved indications, 13.3%; potentially off‐label, 86.7%. Among FDA‐approved users, diagnoses included seizure disorders (86.6%) and post‐herpetic neuralgia (14.7%). Among potentially off‐label users, mutually exclusive pain categories included neuropathic pain only (0.9%), non‐neuropathic pain only (31.3%), both neuropathic and non‐neuropathic pain (66.4%), or neither (1.4%). ^b^Gabapentin, residents with ADRD. FDA‐approved indications, 16.6%; potentially off‐label, 83.4%. Among FDA‐approved users, diagnoses included seizure disorders (88.8%) and post‐herpetic neuralgia (12.6%). Among potentially off‐label users, mutually exclusive pain categories included neuropathic pain only (1.3%), non‐neuropathic pain only (42.1%), both neuropathic and non‐neuropathic pain (54.3%), or neither (2.3%). ^c^Valproic acid, residents without ADRD. FDA‐approved indication, 52.6%, potentially off‐label, 47.4%. Among FDA‐approved users, diagnoses included seizure disorders (58.5%), bipolar disorder (54.1%), and migraine (7.4%). The DBDI is not reported for residents without ADRD. ^d^Valproic acid, residents with ADRD. FDA‐approved indication, 32.8%; potentially off‐label, 67.2%. Among FDA‐approved users, diagnoses included seizure disorders (65.9%), bipolar disorder (44.1%), and migraine (4.9%). Among potentially off‐label users, 31.3% had behavioral symptoms (DBDI > 0).

Among 29,585 valproic acid initiators, 19,173 (64.8%) had no FDA‐approved indication. Off‐label valproic acid initiation was more common among residents with ADRD than those without ADRD (67.2% vs. 47.4%). Among off‐label valproic acid initiators with ADRD, 5475 (28.6%) had behavioral symptoms documented on MDS assessments.

## 4 Discussion

In this national study of long‐stay NH residents, the vast majority of gabapentin initiations (85%) lacked an FDA‐approved indication and were frequently associated with non‐neuropathic pain—a use for which evidence shows limited or no benefit relative to well‐established risks of sedation, falls, and impaired cognition [3]. Nearly two‐thirds of valproic acid initiations were off‐label, with the highest rates among residents with ADRD (67.2%). Notably, a Cochrane review found no benefit of valproic acid for agitation in dementia, while adverse effects—including sedation, gastrointestinal symptoms, and worsened functional status—were significantly more common in treated patients [6, 7].

These findings do not imply that all off‐label prescribing is inappropriate. However, gabapentin and valproic acid may be filling treatment gaps in NH care, where access to multimodal pain management and nonpharmacologic approaches to behavioral symptoms in dementia remains limited [8]. Rising use may also reflect efforts to avoid opioids or antipsychotics amid growing scrutiny of these medication classes, effectively substituting one set of risks for another [1, 9]. That only 28.6% of off‐label valproic acid initiators with ADRD had documented behavioral symptoms raises additional questions about the clinical rationale for many of these prescriptions.

As the Centers for Medicare & Medicaid Services (CMS) and other stakeholders expand monitoring of antiseizure medication use in NHs [10], oversight efforts should distinguish treatment for approved indications from off‐label use. Critically, monitoring alone is insufficient; it must be paired with investment in safer alternatives, including adequate staffing, nonpharmacologic dementia care strategies, and multimodal pain management.

This study has limitations. Diagnostic codes may approximate but cannot confirm prescribing intent, and our approach of using diagnoses before and after initiation likely underestimates the true prevalence of off‐label use. Prescription fills may not reflect medication administration, and fills covered by other insurers would not be captured. Despite these limitations, these findings reveal widespread off‐label use of antiseizure medications and underscore the need for closer clinical review of gabapentin and valproic acid initiation in NH residents, particularly those with ADRD.

## Author Contributions

Lauren B. Gerlach had full access to all the data in the study and takes responsibility for the integrity of the data and the accuracy of the data analysis. Study concept and design: Lauren B. Gerlach, Julie P.W. Bynum, and Donovan Maust. Acquisition, analysis, or interpretation of data: All authors. Drafting of the manuscript: Lauren B. Gerlach, Donovan Maust, Andrew R. Zullo, and Ana Montoya. Critical revision of the manuscript for important intellectual content: All authors. Statistical analysis: Lan Zhang. Obtaining funding: Lauren B. Gerlach. Administrative, technical, or material support: Lauren B. Gerlach. Supervision: Lauren B. Gerlach and Donovan Maust.

## Funding

This work was supported by the National Institute on Aging (K23AG066864, R01AG087073).

## Disclosure

The funding source had no role in the design and conduct of the study; collection, management, analysis, and interpretation of the data; preparation, review, or approval of the manuscript; and decision to submit the manuscript for publication.

## Conflicts of Interest

The authors declare no conflicts of interest.
